# The Feasibility of a Novel Index From a Wireless Doppler Ultrasound Patch to Detect Decreasing Cardiac Output in Healthy Volunteers

**DOI:** 10.1093/milmed/usaa248

**Published:** 2021-01-25

**Authors:** Jon-Émile S Kenny, Andrew M Eibl, Matthew Parrotta, Bradley F Long, Joseph K Eibl

**Affiliations:** Health Sciences North Research Institute, Sudbury, ON P3E 2H2, Canada; Health Sciences North Research Institute, Sudbury, ON P3E 2H2, Canada; Health Sciences North Research Institute, Sudbury, ON P3E 2H2, Canada; Health Sciences North Research Institute, Sudbury, ON P3E 2H2, Canada; Northern Ontario School of Medicine, Sudbury ON P3E 2C6, Canada; Health Sciences North Research Institute, Sudbury, ON P3E 2H2, Canada; Northern Ontario School of Medicine, Sudbury ON P3E 2C6, Canada

## Abstract

**Introduction:**

Early hemorrhage is often missed by traditional vital signs because of physiological reserve, especially in the young and healthy. We have developed a novel, wearable, wireless Doppler ultrasound patch that tracks real-time blood velocity in the common carotid artery.

**Materials and Methods:**

We studied eight healthy volunteers who decreased their cardiac output using a standardized Valsalva maneuver. In all eight, we simultaneously monitored the velocity time integral (VTI) of the common carotid artery (using the ultrasound patch) as well as the descending aorta (using a traditional pulsed wave duplex imaging system); the descending aortic VTI was used as a surrogate for left ventricular stroke volume (SV). Additionally, in a subset of four, we simultaneously measured SV using a noninvasive pulse contour analysis device.

**Results:**

From baseline to peak effect of Valsalva, there was a statistically significant fall in descending aortic and common carotid VTI of 37% (*P* = 0.0005) and 23% (*P* < 0.0001), respectively. Both values returned to baseline on recovery. Additionally, a novel index from the carotid ultrasound patch (i.e., the heart rate divided by the carotid artery VTI) detected a 10% fall in aortic VTI with high sensitivity and specificity (100% and 100%, respectively); this novel index also accurately detected a 10% decrease in SV as measured by the noninvasive SV monitor. The mean arterial pressure, measured by the noninvasive pulse contour device, did not correctly detect the fall in SV.

**Conclusion:**

In summary, a novel index from a wireless Doppler ultrasound patch may be more sensitive and specific for detecting decreased cardiac output than standard vital signs in healthy volunteers.

Over 90% of survivable injuries on the battlefield are attributable to hemorrhage,^1^ and it remains a leading cause of preventable death within the civilian population.^2,3^ Because the hemodynamic consequences of hemorrhage may be hidden by traditional vital signs (e.g., blood pressure, heart rate, respiratory rate),^4,5^ novel methods for the detection, surveillance, and management are timely.

We have developed a novel, wireless, Doppler ultrasound patch that is worn over the common carotid artery and provides beat-to-beat measures such as the area under the velocity time spectrogram (i.e., the velocity time integral or VTI). The VTI gives the distance traveled by the blood through the vessel (in centimeters) per cardiac cycle; the distance traveled is directly related to blood flow through the vessel. Further, the ultrasound patch calculates a novel index which is analogous to the shock index—the Doppler Shock Index (DSI). The DSI is obtained by replacing systolic blood pressure in the denominator with the common carotid artery VTI; preliminary results were presented in abstract form.^6^

Because the common carotid VTI is directly related to blood flow off of the aortic arch, we hypothesize that the VTI will be a more sensitive marker of falling cardiac output. Accordingly, replacing the systolic blood pressure of the shock index with the common carotid VTI should yield a more rapid and sensitive metric for detecting diminished cardiac output.

## METHODS

### Clinical Setting

We recruited eight healthy adult volunteers with no known cardiovascular history and on no regular cardiovascular medications. The study was conducted in the Simulation Lab at Health Sciences North (Sudbury, ON, Canada). Written and informed consent was obtained for all subjects and the study was approved by the Research Ethics Board of Health Sciences North.

### Vital Signs Measurements

In all subjects, heart rate and blood pressure were measured at baseline using palpation and a sphygmomanometer. During the standardized Valsalva maneuver, heart rate was measured using the Doppler ultrasound waveform and blood pressure was measured only in the subjects wearing the noninvasive pulse contour device.

### Standardized Valsalva

All subjects performed a standardized Valsalva maneuver as follows: following 15 seconds of recorded, resting baseline, the subjects were instructed to take a tidal breath in and contract the diaphragm against a manometer placed at the lips to maintain an airway pressure of 20--25 cm H_2_O for 15 seconds; this phase is termed the “inspiratory hold.” Subsequently, the pressure and tidal breath were released to functional residual capacity and this was held for an additional 15 seconds before another breath was taken. Lastly, there was an additional 15 seconds “recovery” phase.

### Descending Aorta VTI Measurement

A duplex ultrasound imaging system (Xario, Toshiba Medical Systems) was employed to acquire simultaneous pulsed wave (PW) Doppler from the descending aorta. The PW Doppler velocity measurement in the descending aorta was measured by a trained sonographer blinded to the velocity data acquired simultaneously from the Doppler ultrasound patch. The duplex system was set up to obtain a view of the descending aorta via the supra-sternal notch, confirmed by identification of the left subclavian artery and pulsatile flow away from a phased array cardiac probe (7.5 MHz), as previously described.^7^ The sample volume was 4 mm long and positioned mid-vessel. The angle of insonation was 0 degrees, as flow was directly away from the probe.

### Carotid VTI Measurement

**FIGURE 1.**
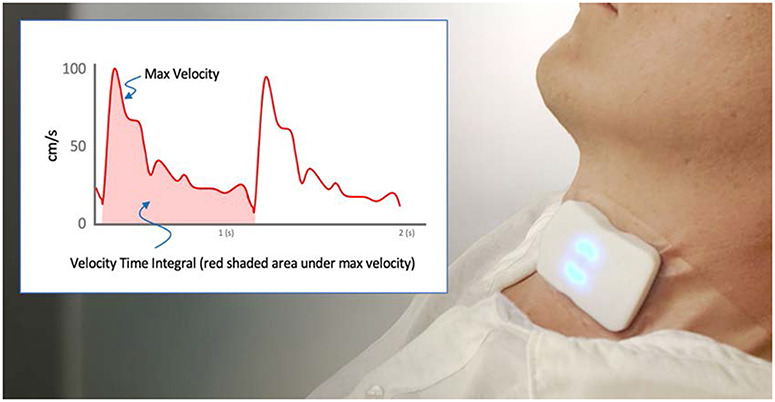
The wireless ultrasound patch on the neck and an illustrated trace from our algorithm and relevant clinical metrics (inset).

The carotid ultrasound patch (Flosonics Medical, Sudbury, ON, Canada) (Fig. 1) was placed by palpation over the carotid artery below the angle of the jaw in an effort to ensure Doppler sampling below the bifurcation. Once an adequate spectrogram signal was visualized in an open-access audio-recording program (Audacity), the protocol was initiated. The maximum velocity of the continuous wave (CW) Doppler waveform was automatically traced using an algorithm based on the approach described by Steinman et al.^8^ The automated maximum velocity estimation for each timepoint in the waveform was used to calculate the VTI as the area under the curve. Fig. 1 shows some of the metrics derived from the CW Doppler patch.

The real-time spectral Doppler signals from the descending aorta (i.e., the duplex, PW system) and the CW Doppler ultrasound patch were fed into a two-channel audio recorder (Roland Corporation, Los Angeles, CA) and then visualized with the Audacity software. This approach ensured synchronous recording of Doppler waveforms from the ultrasound patch and aorta from the PW, duplex imaging system. Additionally, we measured contemporaneous Doppler spectra from the ultrasound patch wirelessly via Bluetooth and correlated it with the data visualized in Audacity.

### Stroke Volume Measurements

A subset of four subjects were monitored with an Food and Drug Administration-approved, noninvasive pulse contour method (Clearsight, Edwards Lifesciences, Irvine, CA) enabling measurements of stroke volume (SV) every 20 seconds. Briefly, Clearsight uses a “volume clamp” system for transducing the digital artery waveform. Using an algorithm, the digital artery waveform is transformed into a brachial artery waveform and then analyzed using pulse contour analysis to derive SV.^9^ A number of studies have evaluated the ability of Clearsight to track changes in cardiac output with agreement values ranging between 84% and 100% compared to a gold standard.^9^ The study protocol did not begin until there was adequate Clearsight signal as measured by the Physiocal metric (i.e., ≥50). The Physiocal metric is a proprietary indicator of adequate signal from the Clearsight device. As recommended by the manufacturer, signal adequacy is acceptable when the Physiocal is at least 50. All subjects were monitored quietly with the Clearsight device for at least 3 minutes before the onset of the standardized Valsalva maneuver.

### Statistical Analysis

For the Doppler metrics, baseline was chosen as time 5--10 seconds of the first 15 seconds of resting baseline (T1). Peak inspiratory hold was defined as the terminal 5 seconds of the 15-second inspiratory hold portion of the Valsalva—labeled as (T2). The return to baseline was defined as the terminal 5 seconds of the 15-second recovery phase—labeled as (T3). Because the Clearsight device updates SV every 20 seconds, we chose resting baseline (T1) as the SV value immediately before the onset of the 15-second resting baseline. The smallest value of SV during the 60-second protocol was taken to be the peak effect of the Valsalva maneuver (T2) while the SV at the end of the 60-second protocol was taken to be the recovery period (T3).

Values obtained for the carotid VTI and descending aortic VTI between baseline and peak inspiratory hold were compared using a two-tailed Student *t*-test. Further, sensitivity and specificity analyses were performed to detect a 10% change in descending aortic VTI between T1 and T3 using the common carotid VTI and the DSI as predictive variables. These data were confirmed using a 10% change in SV in the subset of volunteers who wore the Clearsight device.

## RESULTS

Eight healthy volunteers completed the 60-second standardized Valsalva maneuver. The average age was 34.5 years and 50% were female. The average body mass index, intake heart rate, and blood pressure were 26.5 kg/m^2^, 72 beats per minute, and 119/81 mm Hg, respectively. The Clearsight device was available for four of the eight subjects studied, thus the SV data represent an *n* = 4.

### Hemodynamic Effects during Peak Inspiratory Hold (T1 to T2)

The average fall in SV from T1 to T2 was 27.5 mL or 26% in the 4 subjects in whom SV was monitored. In all eight, there was a statistically and clinically significant fall in aortic VTI and carotid VTI from T1 to T2. The VTI of the aorta fell by 37% (*P* = 0.0005) from T1 to T2; additionally, the carotid VTI fell by 23% (*P* < 0.0001) for the same time period. The DSI (i.e., heart rate divided by the common carotid VTI) increased by 57%, while the traditional shock index rose by only 20%. For the same time period, the mean arterial pressure changed minimally (i.e., +4%). The mean arterial pressure was obtained during the Valsalva in those wearing the Clearsight device; like SV, the MAP is recorded every 20 seconds.

### Hemodynamic Effects of Recovery (T2 to T3)

**FIGURE 2.**
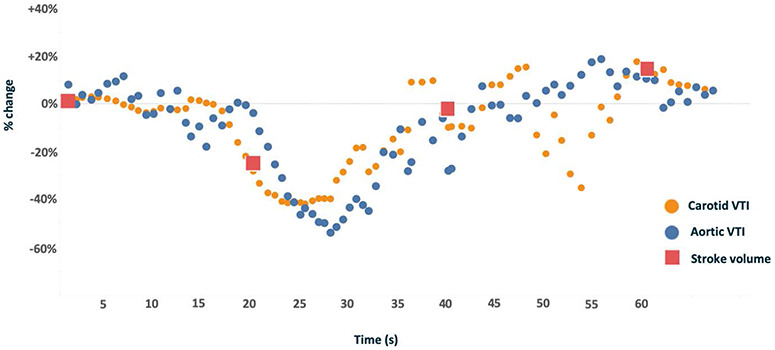
A representative standardized Valsalva maneuver in a single subject showing simultaneously measured carotid and aortic velocity time integral (VTI) as well as from the noninvasive stroke volume monitor. The onset of Valsalva occurs at 15 seconds and is released at 30 seconds; the stroke volume monitor provides an output every 20 seconds.

There was a statistically and clinically significant rise in aortic VTI and carotid VTI upon release of the Valsalva maneuver (+115%, *P* < 0.0001 and\,\, + 32%, *P* = 0.000), respectively. The average rise in SV from T2 to T3 was 37 mL or +10%. The DSI returned to baseline in all subjects during recovery. Fig. 2 illustrates the change in the carotid and aortic VTI—as well as SV—throughout an entire standardized Valsalva maneuver.

### Accuracy Assessments

**FIGURE 3.**
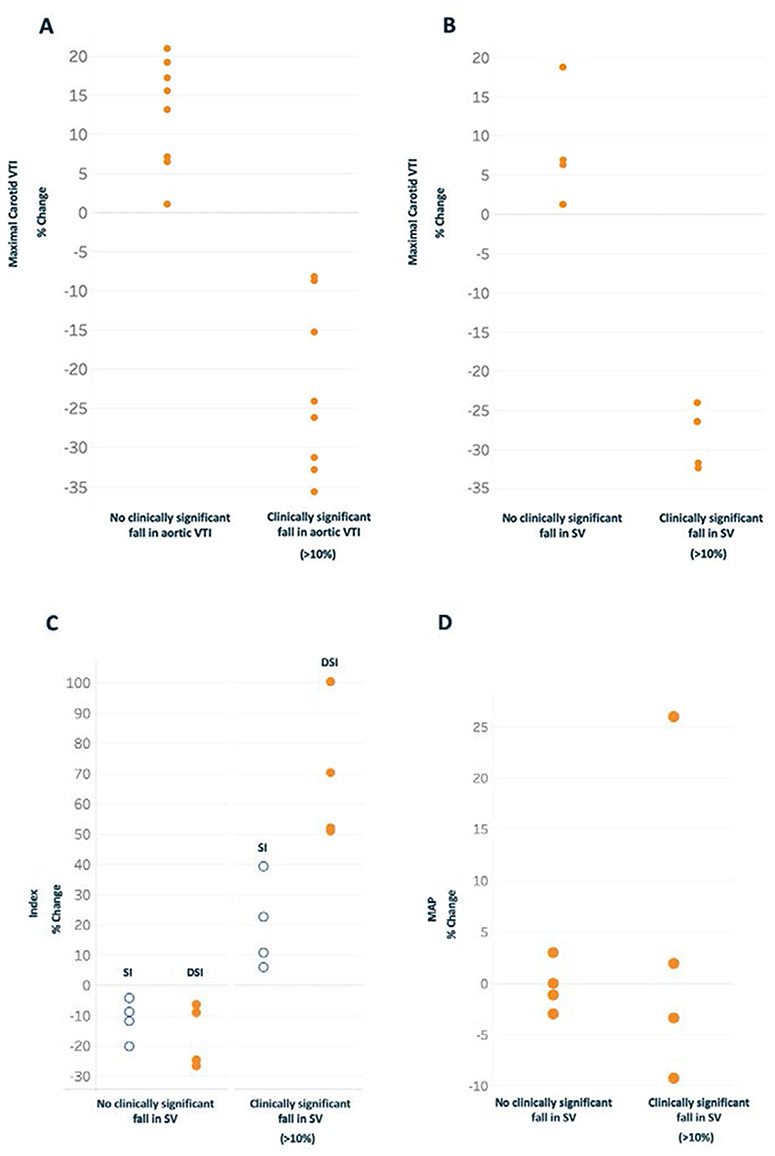
Sensitivity and specificity analyses for the percent change in common carotid velocity time integral (VTI) (*y*-axis) to predict a clinically significant decrease in (A) aortic VTI or (B) stroke volume (SV). (C) The percent change in Doppler shock index (DSI) compared to the traditional shock index (SI) (*y*-axis). Both indices (DSI and SI) achieve perfect separation between subjects with and without a 10% fall in SV yet the separation for the DSI is greater. (D) The same analysis for the mean arterial pressure (MAP).

Figure 3A,B shows the sensitivity and specificity analyses of the carotid VTI for detecting either a 10% change in descending aortic VTI or SV, respectively. Change in the carotid artery VTI achieved perfect sensitivity and specificity for identifying both a significant fall in aortic VTI (*n* = 8, Fig. 3A) and SV (*n* = 4, Fig. 3B). Fig. 3C compares the sensitivity and specificity of the DSI to the traditional shock index for predicting a significant fall in SV. Like carotid VTI, both DSI and traditional shock index achieve perfect separation for detecting a significant fall in SV; however, the separation for the DSI is much greater. Fig. 3D reveals that the MAP could not accurately distinguish a significant fall in SV.

## DISCUSSION

This small, proof-of-concept study demonstrates that a wearable, wireless Doppler ultrasound patch can accurately and immediately detect a fall in both SV and descending aortic VTI. Further, a novel index measured by the ultrasound patch—the DSI—appeared to be more sensitive and specific for identifying a change in cardiac output during a Valsalva maneuver than traditional vital signs.

These findings are clinically significant because the signs of substantial hemorrhage may be subtle.^3^ Indeed, blood pressure can be maintained until upwards of 30% (i.e., roughly 1.5 to 2.0 L) of blood volume is lost.^10^ Additionally, hemorrhage treatment is time-sensitive with the median time from onset to death in hemorrhagic shock being only 2 hours.^11,12^ Accordingly, a novel device and index of resuscitation efficiency may help diagnose, triage, and guide therapy in the field, during transport, and into the hospital. For example, common carotid blood flow measured using a traditional, handheld probe detects central blood volume loss early^13^ and tracks the response to autotransfusion following a passive leg raise.^14^ Accordingly, the Doppler ultrasound pulse and derivatives such as the DSI could refine hemorrhage detection and management.

Interestingly, both the traditional shock index and DSI detected a 10% decrease in SV with perfect sensitivity and specificity in the small subset of subjects who had SV monitored. As seen in Fig. 3C, the absolute separation for the DSI was much greater than that of the traditional shock index—57% versus 10%—respectively. This greater separation suggests, but does not prove, that the DSI is potentially more sensitive and specific if performed on many more subjects. This warrants further investigation.

We have previously shown that the Doppler ultrasound patch described herein accurately tracks rising SV during a maneuver that increased cardiac preload in healthy controls.^15^ The findings of the current investigation extend those results to a paradigm of decreased cardiac preload and cardiac output. The use of Doppler ultrasound of the common carotid artery to infer left ventricular output is supported by a number of previous studies using traditional vascular ultrasound probes.^16–20^ Yet, human factors when using handheld PW Doppler systems can introduce variability in velocity measurement.^21^ The CW Doppler patch described herein may mitigate many of the aforementioned vulnerabilities because of the fixed nature of an adherent transducer.

A number of important limitations of our study must be addressed. First, we measured the VTI and not absolute flow. Because vascular diameter is prone to measurement error and amplified to the second power when calculating area,^22^ we did not measure flow. We chose an aortic VTI cutoff of 10% because a previous report demonstrated that a 10% change in descending aortic blood flow accurately predicted changes in left ventricular output.^23^ We believe that VTI is an adequate surrogate for flow because the fall in descending aortic VTI was mirrored in the common carotid artery as well as by the noninvasive SV monitor. Second, our sample size was relatively small, yet the observed trends were robust and consistent with expected physiology in healthy volunteers; thus, our findings should hold within a relatively healthy adult patient population such as soldiers. Lastly, there has been conflicting data regarding the correlation between SV from noninvasive pulse contour analysis and thermodilution-based methods; however, the ability of the former to track intraindividual change is clinically adequate.^9,24–28^

We believe that the ultrasound patch described has a number of potential applications in military medicine including combat casualty triage and care, monitoring in austere environments, dive physiology as well as detection and prevention of Gz+  associated loss of consciousness. The patch has 2--8 hours of continuous ultrasound scan time. We have used the patch to accurately monitor human physiology in ambient temperatures of −5 to 100°C in a small group of healthy volunteers, though larger studies in more extreme temperatures are planned.

In conclusion, we describe the feasibility and utility of a wireless, wearable Doppler ultrasound patch worn over the common carotid artery. The common carotid VTI and a novel index derived from this new ultrasound patch are able to detect clinically significant changes in central aortic blood velocity and SV. Further study in models of human hemorrhage and active resuscitation protocols are warranted.
